# Pulmonary embolism post-Covid-19 infection: physiopathological mechanisms and vascular damage biomarkers

**DOI:** 10.1007/s10238-023-01150-w

**Published:** 2023-08-03

**Authors:** Luigi Petramala, Francesca Sarlo, Adriana Servello, Silvia Baroni, Marianna Suppa, Francesco Circosta, Gioacchino Galardo, Orietta Gandini, Luca Marino, Giuseppe Cavallaro, Gino Iannucci, Antonio Concistrè, Claudio Letizia

**Affiliations:** 1 Department of Translational and Precision Medicine, “Sapienza” University of Rome, Rome, Italy; 2 UOC Chimica, Biochimica e Biologia Molecolare Clinica, Fondazione Policlinico Universitario A. Gemelli I.R.C.C.S., Rome, Italy; 3 Emergency Medicine Unit, Department of Emergency-Acceptance, Critical Areas and Trauma, Policlinico “Umberto I”, Rome, Italy; 4 Scienze Biotecnologiche di Base, Cliniche Intensivologiche e Perioperatorie, Università Cattolica del Sacro Cuore, Rome, Italy; 5 Department of Clinical, Internal, Anesthesiological and Cardiovascular Sciences, “Sapienza” University of Rome, Rome, Italy; 6 Department of Molecular Medicine, “Sapienza” University of Rome, Rome, Italy; 7 Department of Mechanical and Aerospace Engineering, “Sapienza” University of Rome, Rome, Italy; 8 Department of Surgery Pietro Valdoni, Sapienza” University of Rome, Rome, Italy

**Keywords:** Pulmonary embolism, Post-COVID-19, sST2 biomarker, Endothelium damage

## Abstract

Covid-19 infection is characterized by several acute complications, as well long-term sequelae, mostly sustained by endothelial dysfunction; several studies show that complications as pulmonary embolism (PE) are described both in the acute phase and after negativization. Aim of research was to evaluate anthropometric, bio-humoral, instrumental parameters in a group of patients affected by PE after recent Covid-19 infection compared to PE patients without previous Covid-19 infection. We enrolled 72 consecutive patients (35M, 37F) with acute PE, distinguished in relation to previous acute Covid-19 infection: 54 pts without previous acute Covid-19 infection and 18 pts with previous Covid-19 infection within negativity at least 2 months before PE diagnosis; 44 healthy subjects (21M, 23F) were recruited as control group. Patients who had previously developed Covid-19 needed hospitalization in high percentage (84%); this group showed significantly higher prevalence of diabetes mellitus than Covid-19-free PE patients, reduced serum levels of C-reactive protein, sST2 and PESI score. In post-Covid-19 PE group, we observed higher mean IMPROVE risk score, whereas in Covid-19-free group lower P/F ratio, higher radiological severity, and worse PESI score and severity index**.** Covid-19 infection affects not just the lung parenchyma but also other organs; endothelial damage plays pivotal role in long-term alterations; in high thrombotic risk group (recent hospitalization due to acute Covid-19 infection), we have described thrombotic complications characterized by persistent prothrombotic state after recovery, highlighted by well-known markers as PCR and D-Dimer as well as novel vascular marker (sST2).

## Introduction

Short- and long-term complications of SARS-CoV2 virus infection are an important health issue of management of patients healed by Covid-19 infection. At present time, considerable efforts are spent to investigate the pathophysiological mechanism underlying the multiple clinical scenarios observed. The systemic involvement of the virus infection lets possible complications on almost all organs and tissues. Beyond the acute phase characterized by pulmonary damage, both parenchymal and vascular, the most dangerous and potentially lethal witnessed sequelae involve cardiac (ischemia, arrhythmias, myocarditis, cardiomyopathies) [1, 2], pulmonary [3, 4] neuropsychiatric [5], gastrointestinal and hepatic [6], nephrological [7–9], vascular manifestations [10].

In particular, endothelium dysfunction is acknowledged as a pivotal role in acute phase as well as in long-term complications. Due to the numerous physiological functions of endothelium (homeostasis, barrier integrity and permeability, inflammation and oxidative stress control), the consequences of its damage can lead to important clinical consequences. The healthy endothelium maintains a dynamic equilibrium between the mechanisms underlying vasodilation, thrombolysis, inflammation, platelet aggregation and oxidation processes [11, 12]. Endothelial-related coagulation disorders can produce an hypercoagulative and pro-thromboembolic state with life-threatening thrombotic events, like pulmonary embolism (PE) and disseminated intravascular coagulation (DIC); although experience and knowledge on pathological mechanisms progressively increase, the specific molecular processes triggered by the virus invasion are still object of analysis. Pro-thrombotic attitude is driven by two complementary mechanisms: an hypercoagulative state directly affecting thrombosis and thromboembolism in large vessels and endothelial damage linked to microvascular dysfunction [3, 13].

Since the first cases of Covid-19 infection, the main hematologic parameters underlying a coagulopathy have been found altered and directly connected to the severity of the disease. Fibrinogen, D-dimer, thrombin, factors V and VIII are now recognized as clinical markers adopted to evaluate the risk stratification and prognosis of complications, whereas inflammation parameters (i.e., ferritin, IL-6) add valuable information to evaluate endothelial injury [14].

Recently, high interest was given also to the ST2 biomarker, as a possible laboratory parameter to assess the severity of the thromboembolic disease [15]. The soluble isoform sST2 is involved in the molecular signaling pathways related to the interleukin IL-33. In particular, the ST2 protein is biologically found as two possible isoforms: a transmembrane protein ST2-L that, as member of the IL-1 receptor superfamily, binds to IL-33 to activate anti-inflammatory and anti-fibrotic molecular processes. The soluble form sST2 has a decoy receptor function by linking with the free IL-33 and inhibiting its action. The role of sST2 as a biomarker has been studied by several authors in different diseases [16, 17]. Initially studied to evaluate heart failure and atrial fibrillation prognosis in chronic conditions, recently it was considered also in acute conditions of heart failure and pulmonary embolism (PE), beyond for diagnostic purposes, also for prognostic role. Regarding pulmonary embolism, a recent study confirmed its potential usefulness in clinical practice to stratify the risk and to assess the disease severity [15].

Aim of this research was to evaluate anthropometric, bio-humoral and instrumental parameters in a group of patients affected by PE after recent Covid-19 infection compared to PE patients without previous Covid-19 infection; in addition to the well-known bio-humoral and clinical parameters of PE severity, in these patients we evaluated new vascular markers such as the plasma levels of sST2, evaluating possible correlations between these parameters and indices and the PE severity.

## Materials and methods

This observational cohort study included 72 consecutive patients (35 male, 37 females; mean age 66 ± 3.7 years) admitted to the Emergency Medicine Department, Policlinico Umberto I Hospital—Rome between 1 October 2021 and 31 July 2022 who had a diagnosis of PE at hospital admission. PE was diagnosed by spiral CTPA in all patients with suspected PE by clinical and laboratory evaluation. We have distinguished these patients into two groups in relation to the possible previous presence of an acute Covid-19 infection: 54 pts (30 male; 24 female; mean age 70 ± 3.4 years) had not presented a previous acute Covid-19 infection (Covid-19-free PE); 18 pts (6 male; 12 female; mean age 64.3 ± 5.3 years) reported a previous Covid-19 infection within negativity at least 2 months before PE diagnosis (mean time from Covid-19 negativization: 16 ± 3.8 weeks), without previous diagnosis of PE or other thromboembolic complication (post-Covid-19 PE). Forty-four healthy subjects (21 male, 23 females; mean age: 62 ± 7.2 years) with no previous history of disease and normal physical examination findings were recruited into the control group and included in the study. Data were collected on the clinical and laboratory findings of the patients at diagnosis including the complete blood count (CBC), lactate dehydrogenase (LDH), C-reactive protein (CPR), ferritin, d-dimer, high-sensitivity troponin T (TNT-hs), prothrombin time (PT), activated partial thromboplastin time (aPTT), creatine phosphokinase (CPK), electrolytes, liver enzymes, arterial blood gas values, the presence of underlying diseases or predisposing factors for PE. In addition to these parameters, sST2 serum concentration was measured in all patients. All consecutive patients with suspicious clinical symptoms associated with PE admitted to our Emergency Medicine Department were prospect evaluated. Blood samples were taken from the brachial vein using vacutainer tubes without anticoagulants. The pulmonary embolism severity index (PESI) was used for clinical scoring to stratify the PE enrolled patients into five different risk classes [18]. Based on the severity, arterial filling defects detected by CTPA three subgroups have been defined (massive, sub-massive and segmental PE). The exclusion criteria of the patient group were as follows: acute ischemic disease such as acute cerebrovascular disease, acute coronary syndrome, acute peripheral arterial occlusion, or acute intestinal ischemia; advanced heart failure, chronic kidney disease, liver cirrhosis, or inflammatory diseases; or refusal to participate in the study.

As previously validated [19], we used sST2 SEQUENT-IA™ kit, a turbidimetric immunoassay (Critical Diagnostics, CA, USA) implemented on ADVIA Chemistry XPT System (Siemens Healthcare Diagnostics, USA) according to procedure provided by the manufacturer. The analytical performance of the assay was evaluated according to the Clinical and Laboratory Standards Institute (CLSI) EP15-A3 guidelines [20].

Statistical analysis was performed using SPSS 28.0 for Mac OS (SPSS, Chicago, IL, USA). All data were expressed as means ± standard deviation (SD). Power analysis was performed to determine the sample size, alpha = 0.05, and the power of the test was calculated as 0.7. Differences between means were assessed by the Student’s t test or the Mann–Whitney U test in non-normally distributed data for two-sample comparison, or by one-way analysis of variance (ANOVA) applying the Fisher least significant difference post hoc test for multiple comparisons. Chi-squared statistics were used to assess differences between categorical variables. Relationships between continuous variables were assessed calculating the Pearson correlation coefficient or the Spearman rank correlation coefficient when appropriate.

## Results

Anthropometric, clinical characteristics, and blood gas parameters of patients are reported in Table 1. There were no differences between PE patients’ groups regarding age and sex, as well as systolic blood pressure (SBP) and diastolic blood pressure (DBP). Overall, PE patients had higher prevalence of diabetes and oncologic disease than controls (7.1% vs. 2.9%; *p* < 0.001; 24.5% vs. 0%; *p* < 0.001, respectively). Moreover, both PE groups showed increased mean heart rate (HR) respect controls (100 ± 26.2 bpm vs. 75 ± 11 bpm, *p* = 0.03). Compared with the healthy subjects, we found significantly increased serum levels of D-dimer in the PE group (3632 ± 221 ng/mL vs. 356 ± 98 ng/mL, *p* < 0.001), as well as high-sensitivity troponin T (TNT-hs) (0.039 ± 0.0075 μg/L vs. 0.009 ± 0.003 μg/L, *p* = 0.04), fibrinogen (475.5 ± 20.1 mg/dL vs. 164 ± 49 mg/dL, *p* < 0.001), and C- reactive protein (CRP) (8.01 ± 4.87 vs. 1.2 ± 0.99, *p* = 0.05). The analysis of biomarker levels in the whole cohort revealed that the PE patients had significantly higher levels of sST2 compared with the healthy subjects (85.86 ± 18.2 ng/mL vs. 17.3 ± 0.36 ng/mL, *p* < 0.001). Compared with controls, in the PE patients the blood gas data showed a lower ratio of arterial oxygen partial pressure to fractional inspired oxygen (P/F ratio) (271.5 ± 99.8 vs. 403 ± 28, *p* = 0.02) and arterial CO2 (36.76 ± 2.51 vs. 40.3 ± 3.2 *p* = 0.02), as well as increased values of serum lactate (1.94 ± 0.51 mmol/L vs. 0.74 ± 0.04 mmol/L, *p* < 0.001); consequently in the PE group, there was a higher percentage of oxygen administered than in the control subjects (34.91 ± 3.12 mmol/L vs. 21 ± 0 mmol/L, *p* = 0.02).

**Table 1:** Anthropometric, biochemical and blood gas parameters in overall patients enrolled, and in PE patients Covid-19-free and EP post-Covid-19

	Controls (n = 44)	PE patients (n = 72)	p-value Controls versus overall PE	PE post-Covid (n = 18)	PE Covid-free (n = 54)	p-value PE post-Covid versus PE Covid-free
Age (years)	62 ± 7.2	66 ± 3.7	0.71	64.3 ± 5.3	70 ± 3.4	0.251
Men (%)	47.7	48.6	0.9	33.3	57.4	0.150
Hypertension (%)	28	41	0.57	33.3	43.5	0.376
Diabetes (%)	2.9*	7.1	** < 0.001**	16.6	4.3	**0.04**
HF/Cardiomyopathy (%)	3.2	1.21	0.567	0	1.8	0.850
Obesity (%)	15.5	42.5	0.35	27.7	46.3	0.102
Oncologic (%)	0*	24.5	** < 0.001**	16.6	30.4	0.061
COPD (%)	18%	28%	0.0670	33%	27%	0.325
SBP (mmHg)	129 ± 13.1	135 ± 10.9	0.12	137.7 ± 6.5	133.1 ± 6.6	0.887
DBP (mmHg)	62 ± 8.8	78 ± 14.37	0.54	81.8 ± 5.9	73 ± 3.5	0.467
HR (bpm)	75 ± 11*	100 ± 26.2	**0.03**	93.7 ± 8.9	103.86 ± 5.7	0.477
RR (bpm)	16 ± 7	19.34 ± 6.54	0.76	16.8 ± 2.4	19.7 ± 3.2	0.213
D-dimer (< 550 ng/mL)	356 ± 98*	3632 ± 221	** < 0.001**	3149 ± 737	3759 ± 238	0.083
Creatinine (1.2 mg/dL)	0.83 ± 0.19	1.08 ± 0.23	0.075	1.43 ± 0.8	0.96 ± 0.07	0.068
TNT-hs (< 0.0014 μg/L)	0.009 ± 0.003*	0.039 ± 0.0075	**0.04**	0.026 ± 0.013	0.045 ± 0.009	0.130
Fibrinogen (200–400 mg/dL)	164 ± 49*	475.4 ± 20.1	** < 0.001**	485 ± 33	461 ± 23	0.756
C- reactive protein(< 0.5 mg/dL)	1.2 ± 0.99*	8.01 ± 4.87	**0.05**	5.72 ± 1.94°	10.24 ± 2.06	**0.029**
PT-INR (0.8–1.2)	0.98 ± 0.2	1.21 ± 0.082	0.075	1.05 ± 0.03	1.12 ± 0.04	0.678
aPTT (0.8–1.2)	0.73 ± 0.36	0.94 ± 0.052	0.2	0.94 ± 0.04	0.91 ± 0.04	0.456
sST2 (ng/mL)	17.3 ± 0.36*	85.86 ± 18.2	** < 0.001**	25.04 ± 3.6°	101.9 ± 19.8	**0.033**
PESI score	35 ± 18.3*	118.1 ± 9.8	** < 0.05**	67.8 ± 7.7°	134 ± 6.7	**0.002**
Severity PE and Risk of early death						
High (%)	0	0	n.d	0	0	n.d
Intermediate–high (%)	0	21	< 0.001	0	13	< 0.001
Intermediate–low (%)	0	69	< 0.001	66	70	0.583
Low (%)	0	10	< 0.001	33	17	0.089
Improve risk score (mean)	0.45 ± 0.1*	1.99 ± 0.3	**0.01**	2.86 ± 0.4°	1.68 ± 0.2	**0.012**
Improve risk score (% > 4 or 2 plus D-Dimer > 2ULN)	0%*	66.1%	**0.01**	85%°	59.1%	**0.05**
Previous Hospital admission (covid-19 related)	0%	84%	0.564	84%°	0%	**0.003**
pH	7.39 ± 0.19	7.46 ± 0.16	0.8	7.50 ± 0.04	7.45 ± 0.02	0.395
pO_2_ (mmHg)	90.5 ± 6.2	88.7 ± 4.1	0.2	80 ± 5	90 ± 4.4	0.231
pCO_2_ (mmHg)	40.3 ± 3.2*	36.76 ± 2.51	**0.025**	41.07 ± 2.5	34.47 ± 1.7	0.197
FiO_2_ administered (%)	21 ± 0*	34.91 ± 3.12	**0.02**	29.8 ± 4.3	36.45 ± 2.7	0.237
SpO_2_ (%)	96.1 ± 1.5	95.7 ± 1.04	0.15	95.4 ± 2.1	95.04 ± 1.9	0.981
P/F ratio	403 ± 28*	271.5 ± 99.8	**0.02**	308 ± 59°	261 ± 26	**0.042**
Lactate (mmol/L)	0.74 ± 0.04*	1.94 ± 0.51	** < 0.001**	1.2 ± 0.21	1.96 ± 0.47	0.257

*COPD* Chronic obstructive pulmonary disease, *SBP* systolic blood pressure, *DBP* diastolic blood pressure, *HR* Heart rate, *RR* respiratory rate, *TNT*-hs high-sensitivity Troponin T, *PT-INR* prothrombin time—international normalized ratio, *aPTT* activated partial thromboplastin time, *PESI* pulmonary embolism severity index, Severity PE and Risk of early death in relation to 2019 ESC Guidelines for the diagnosis and management of acute pulmonary embolism

Bold stays for statistical significant (*p* < 0.05) cases

Overall, PE patients had also increased PESI score (118.1 ± 10.6 vs. 35 ± 18.3, *p* < 0.001), mean IMPROVE risk score (1.99 ± 0.3 vs. 0.45 ± 0.1, *p* = 0.01) and percentage of IMPROVE mean score more than 4 or more than 2 plus d-dimers over 2ULN than controls (66.1% vs. 0%, *p* = 0.01, respectively).

The comparison between the two PE groups showed significantly higher prevalence of diabetes mellitus in post-Covid-19 PE group than Covid-19-free PE patients (16.6% vs. 4.3%, *p* = 0.04), whereas Covid-19-free PE group showed slightly higher prevalence of oncologic diseases (30.4% vs. 16.6%, *p* = 0.061) respect to post-Covid-19 PE group. Compared with Covid-19-free PE subjects, in post-Covid-19 PE subjects we found significantly decreased serum levels of C-reactive protein (5.72 ± 1.94 mg/dL vs. 10.24 ± 2.06 mg/dL, *p* = 0.029), sST2 (25.04 ± 3.6 ng/mL vs. 101.9 ± 19.8 ng/mL, *p* = 0.033) and PESI score (67.8 ± 7.7 vs. 134 ± 6.7, *p* = 0.002). Remarkably, patients who had previously developed Covid-19 showed mild form of disease (lung involvement was less than 50%), needing hospitalization in high percentage (84%); during the hospitalization, overall patients were treated with oxygen supplementation (average oxygen 31 ± 4%) and low-molecular-weight heparin (LMWH 4.000UI/die); the mean length of hospitalization was 9.3 ± 2.7 days. Interestingly, Covid-19-free group showed an higher percentage of patients (13%) with intermediate–high risk of early death (evaluated through ESC guidelines), with respect PE post-Covid (0%; *p* < 0.001).

**Fig. 1:**
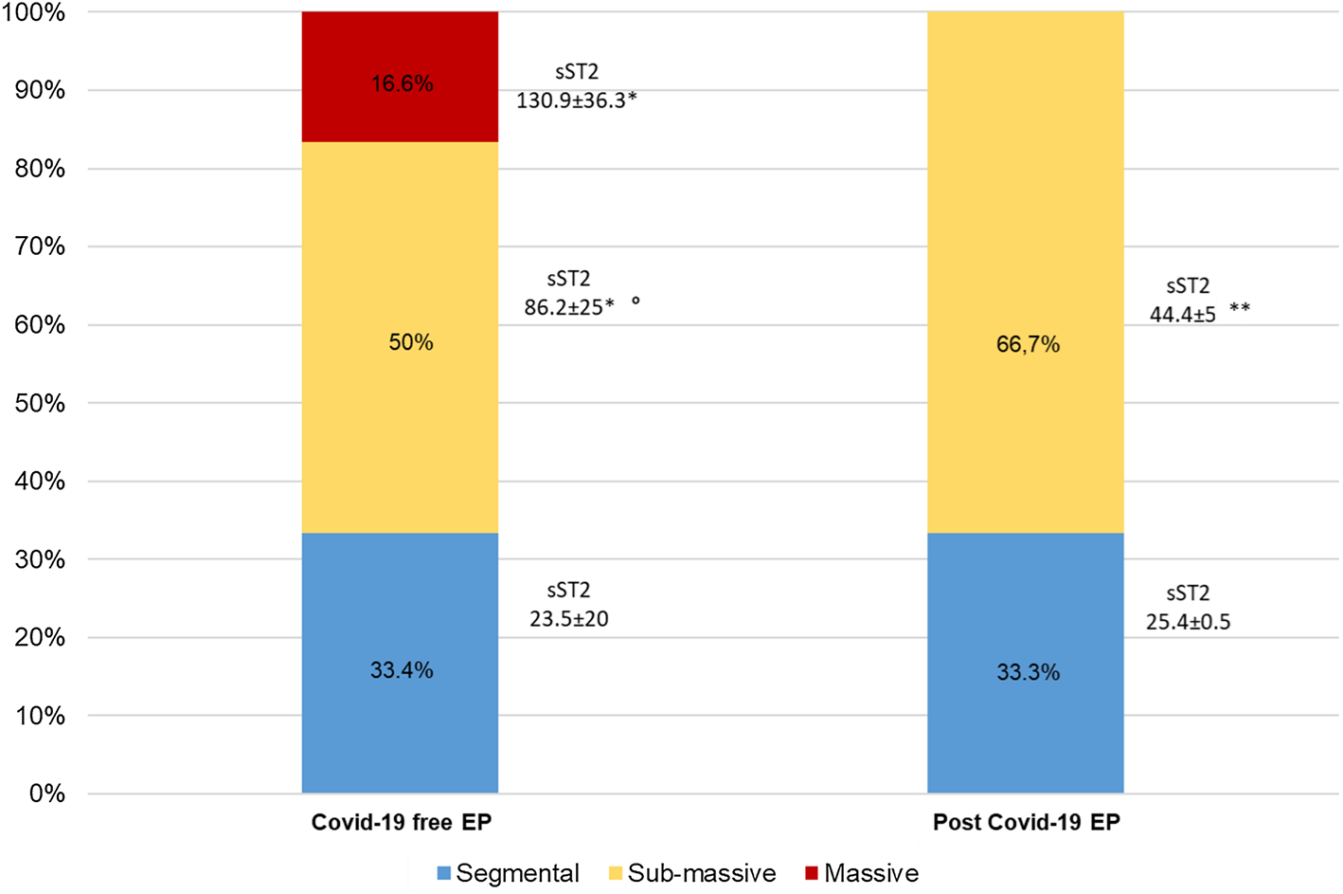
Radiological severity of Pulmonary Embolism dividing EP Covid-19-free and EP post-Covid-19; values of sST2 behaviors in specific group. *p < 0.01 versus Segmental group; °*p* < 0.01 versus Sub-massive group post-Covid-19 EP

Thus, in post-Covid-19 PE group we have observed higher mean IMPROVE risk score (2.86 ± 0.4 vs. 1.68 ± 0.2, *p* = 0.012) as well as higher percentage of subjects with IMPROVE risk score > 4 or > 2 plus d-dimers > 2ULN (85% vs. 59.1%, *p* = 0.05). The blood gas data showed a lower P/F ratio in the Covid19-free patients compared to the post-Covid-19 PE group (261 ± 26 vs. 308 ± 59, *p* = 0.042). Figure 1 shows vascular involvement by angiographic study in EP patients; in Covid-19-free EP group, we observed higher radiological severity respect post-Covid-19 EP group (massive involvement 16.6% vs. 0%; *p* = 0.05); the sST2 values were greater in each group in massive/submassive involvement with respect to subsegmental involvement, but interestingly, in post-Covid-19 EP group sST2 behaviors were lower respect Covid-19-free EP group in those patients with submassive vascular involvement (44.4 ± 5 vs. 86.2 ± 25 ng/mL, *p* < 0.01).

In Table 2, we have reported data of blood gas and thrombotic load in PE patients Covid-19-free and PE post-Covid-19, distinguished in relation to severity PE and risk of early death according to ESC Guidelines for the diagnosis and management of acute PE. As regard, we found that PE Covid-19-free group showed lower values of pCO_2_ in “Low Risk group” (35.7 ± 3.5 mmHg) and in “Intermediate-Low group” (35.9 ± 5.8 mmHg) respect with post-Covid-19 group (43 ± 5.5 mmHg and 40.8 ± 5.5 mmHg, respectively; *p* < 0.05).

**Table 2:** Blood gas parameters and thrombotic load in PE patients Covid-19-free and PE post-Covid-19, distinguished in relation to Severity PE and Risk of early death in relation to 2019 ESC Guidelines for the diagnosis and management of acute pulmonary embolism

	PE post-Covid (*n* = 18)			PE Covid-free(*n* = 54)			*p*-value
	Intermediate–high	Intermediate–low	Low	Intermediate–high	Intermediate–low	Low	
pH	–	7.43 ± 0.03	7.53 ± 0.18	7.44 ± 0.07	7.46 ± 0.05	7.44 ± 0.06	NS
pO_2_ (mmHg)	–	83.5 ± 4.5	88.5 ± 6.7	85.5 ± 7.5	87.38 ± 0.05	93.0 ± 3.5	NS
pCO_2_ (mmHg)	–	40.8 ± 5.5	43 ± 5.5	32 ± 5.5	35.9 ± 5.8*	35.7 ± 3.5**	* 0.05 **0.05
FiO_2_ administered (%)	–	31 ± 6	24.5 ± 4.9	46.67 ± 5.7	37.9 ± 13.5	22.75 ± 3.5	NS
SpO_2_ (%)	–	94.7 ± 7.1	95.1 ± 8.3	94 ± 2.5	95.2 ± 4.1	96 ± 3.5	NS
P–F ratio	–	333 ± 44	370 ± 70	212 ± 20	278 ± 50	399 ± 36	NS
Lactate (mmol–L)	–	1.4 ± 0.7	0.9 ± 1.1	2.76 ± 2.0	1.72 ± 1.53	1.25 ± 0.17	NS
D-dimer (< 550 ng–mL)	–	4427 ± 357	3823 ± 154	4247 ± 115	3683 ± 400	3642 ± 654	NS

*Intermediate–low PE post-Covid versus Intermediate–low PE Covid-free

**Intermediate–low PE post-Covid versus Intermediate–low PE Covid-free; NS: not significant

**Fig. 2:**
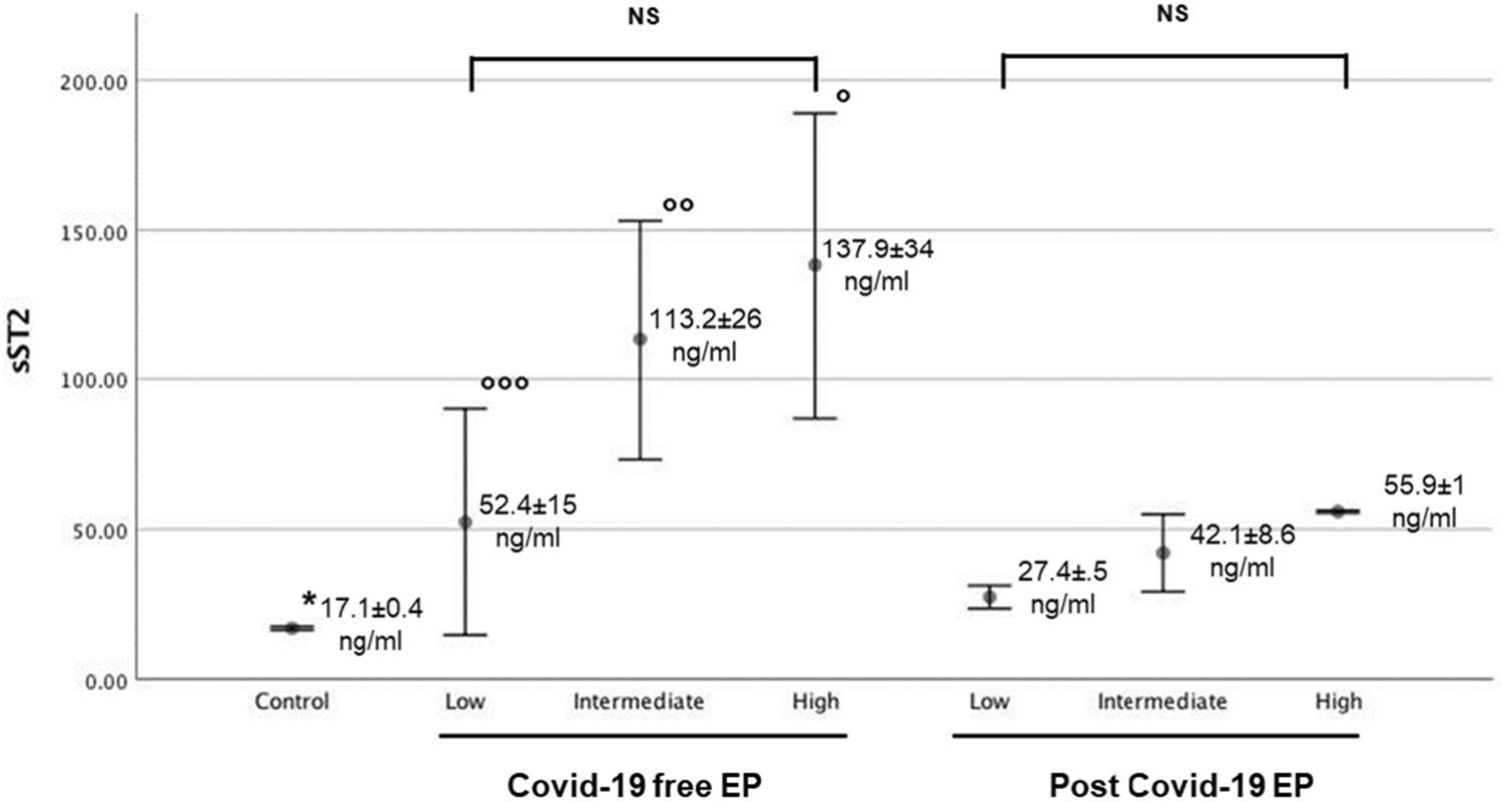
sST2 behaviors (mean ± sd) in the control group and in the PE groups (EP Covid-19-free and EP post-Covid-19), distinguished according to PESI score: low (I–II class, < 85), intermediate (III class, 86–105) and high IV–V class, > 106). *p < 0.01 versus all EP Covid-19-free and EP post-Covid-19; °p: 0.03 versus high—EP post-Covid-19; °°p: 0.01 versus Intermediate—EP post-Covid-19; °°°p: 0.09 versus low—EP post-Covid-19

Moreover, in Fig. 2 we reported the behavior of sST2 according to PESI score in all studied groups; control group showed lower sST2 levels with respect to all EP patients (17.1 ± 0.4 ng/mL; *p* < 0.01). Covid-19-free EP group showed higher sST2 levels in relation to PESI severity for all 3 classifications (low: 52.41 ± 15 ng/mL vs. 27.45 ± 2.5 ng/mL; *p* = 0.05, intermediate: 113.2 ± 26.6 ng/mL vs. 42.1 ± 8.6 ng/mL; *p* = 0.01, high: 137.9 ± 34 ng/mL vs. 55.9 ± 1 ng/mL; respectively, *p* = 0.03).

## Discussion

Several retrospective and prospective studies show higher incidence of adverse events, with odds ratio (OR) 1.6 for post-discharge complications after Covid-19 hospitalization compared Covid-19 outpatients, and significant mortality rate (4.8%) up to 6/12 weeks after 45 days hospital discharge, especially in acute Covid-19 infection requiring intensive/subintensive care admission [21]. As regard it, in a relevant multicenter study the rate of death was 3.3% after 45 days from in discharge, mainly related to myocardial infarction, heart failure, and stroke [22].

On the other hand, several studies show that in addition to increased thromboembolic risk (OR 2.5 for thrombosis at day 30), in patients discharged after hospitalization for Covid-19, there is also higher risk for non-fatal bleeding (OR for major hemorrhage 0.7, OR for non-major bleeds 2.9). Therefore, it becomes pivotal to stratify the balance between thrombotic and hemorrhagic risk in relation to clinical and biomolecular risk factors, as severity of the infection, age, prior venous thromboembolism (VTE), admission in intensive care unit (ICU), chronic kidney disease, peripheral arterial disease, carotid occlusive disease, coronary artery disease, IMPROVE VTE risk score > 4 or > 2 plus an elevated D-dimer (> 2X ULN) [23].

Previously evaluated as a useful tool in non-Covid-19 populations, the IMPROVE VTE risk model has been validated in order to assess risk for VTE and suggests the utility of antithrombotic therapy in large studies conducted on patients hospitalized for Covid-19, highlighting VTE rate 5.30% with scores more than 4 [24].

Observational studies conducted in the early stages of the epidemic showed that in outpatients non-previously hospitalized, the rates of PE on CT imaging in emergency departments were about 20%, and the first one autoptic studies found pulmonary embolism as the probable cause of death in patients never hospitalized who died at home from Covid-19 [25, 26].

Used even just at prophylactic dose in patients hospitalized during acute phase of Covid-19 infections, low-molecular-weight heparin (LMWH) has been shown to significantly reduce the mortality rate. In particular, beyond well-established effect on blood-coagulation, the LMWHs have been recognized to have anti-inflammatory properties, consisting of inhibiting adhesion, chemotaxis, activation or proliferation of leukocyte, allosteric binding site on the T-cell receptor which prevents T-cell receptor activation, reduction in interferon-gamma secretion, interleukins and TNF-α [27].

The decision to use antithrombotic therapy after discharge must necessarily be based on the assessment of the severity of the previous Covid-19 infection and its complications, and possible pre-existing cardiovascular and metabolic risk factors.

As regard it, the multicentre-randomized trial (MICHELLE Trial) [28] was conducted administrating new oral anticoagulants (NAO) (rivaroxaban 10 mg/day) for 35 days after hospital discharge in high-risk discharged patients (52% treated in intensive care unit, 62% with IMPROVE score > 2 plus elevated D-dimer levels, 38% with IMPROVE score > 4). This trial showed that oral thromboprophylaxis improved clinical outcomes (symptomatic, asymptomatic or fatal venous thromboembolism) compared with no extended thromboprophylaxis (relative risk 0·33; *p* = 0·0293), without major bleeding.

On the other hand, a large multicenter study ACTIV-4B [29] recently was conducted in North America in outpatients with stable and symptomatic infection of Covid-19 without requirement of hospitalization; this study has evaluated the rates of composite outcome (all-cause mortality, symptomatic venous or arterial thromboembolism, myocardial infarction, stroke, or hospitalization for cardiovascular or pulmonary cause) after treatment with aspirin (81 mg once daily), apixaban (2.5 mg twice daily), apixaban (5.0 mg twice daily), or placebo, showing low rate of composite outcomes after 45 days of observation, and no significant differences between the active groups and the placebo group. The results of this study must be evaluated in relation to the low percentage of cardiovascular risk factors observed in this population (prevalence of arterial hypertension 33–40%, diabetes mellitus 15–22%, D-Dimer > 2 ULN 9–14%).

Similar results were obtained in two relevant studies; the OVID Study has evaluated the useful of administration of subcutaneous enoxaparin 40 mg once daily for 14 days versus standard of care (no thromboprophylaxis), not detecting differences regarding primary outcome (hospitalization and all-cause death within 30 days); as previous study, in this casuistry the study group has a low rate of cardiovascular risk (arterial hypertension 24.5%; diabetes mellitus 8%; chronic heart failure < 1%; previous malignancy 4.5%) [30]; same results were observed in the multicenter ETHIC Study, conducted in a population of outpatient setting plus at least one risk factor for severe disease (diabetes 31%; active cancer 1%, vascular disease 16%; heart failure < 1%; hypertension 70%; previous venous thromboembolism < 2%) using enoxaparin 40 mg once or twice daily on the basis of bodyweight for 21 days [31].

In our study, an high percentage of patients who presented PE after discharge for Covid-19 had required a previous hospitalization for acute infection; compared to patients without previous Covid-19 infection, these patients presented a greater thrombotic risk terms of IMPROVE risk score (both mean value or percentage of patients with a value greater than 4 or 2 plus D-Dimer > 2ULN).

Moreover, compared to PE patients Covid-19-free, in PE patients after Covid-19 infection our study showed lower severity regarding both vascular extension and respiratory impairment of thromboembolic condition (lower PESI score, lower vascular involvement on CT scan, lower levels of PCR), as well as less severe pathophysiological consequences (better P/F ratio and lower need for oxygen), describing a phenotype characterized by increased and persisting prothrombotic condition after healing from acute Covid-19 infection.

In this regard, in a recent study we have evaluated the behaviors of soluble ST2 (sST2), a cardiovascular injury-related biomarker currently recognized in the stratification of mortality in heart failure as well in acute infection diseases as Covid-19 pneumonia, in patients affected by acute PE. In this study, we have shown that sST2 levels are related to the extension of PE and its pathophysiological complications [15].

In this study, we found that patients with PE after healing from Covid-19 showed elevated levels of sST2 compared to the healthy population but reduced compared to patients with acute PE not related to previous Covid-19 infection. These data support the thesis of a sub-acute prothrombotic state due to endothelial damage after the Covid-19 infection, especially in those subjects who required hospitalization for acute infection (84% was previously hospitalized for Covid-19 infection) or with previous cardiovascular diseases (i.e., higher prevalence of diabetes). Interestingly, in our series we found that Covid-19-free group had worse indices of severity and risk of early death than patients with previous Covid-19 infection; in particular in Covid-19-free group, we found higher frequency of cancer in the anamnesis, and higher risk of mortality according to the ESC stratification [32]; on another hand, PE patients with previous Covid-19 infection appear to have less severe forms of PE but had higher frequency of diabetes, relevant risk factor for persistence and recurrence of PE, confirming that diabetes is either an important risk factor for recurrence and Post-Pulmonary Embolism Syndrome, as well as a risk factor for complications after Covid-19 infection.

The Covid-19-related mechanisms favoring the onset of thromboembolic events are several, acting through many ways, overall maintaining endothelial dysfunction, regional and systemic inflammation, hemostatic disorders and prothrombotic state [27, 33]. Several cells and mediators of innate and adaptive immunity, vessel walls and other epithelial barriers are involved in development of Covid-19-related complications [34]. Lung tissue is widely infiltrated by neutrophils and megakaryocytes, activated platelets, creating proinflammatory and prothrombotic conditions necessary for local intra- and extra-vascular clots made of platelets and fibrin [35].

While diffuse alveolar damage characterized by impermeable hyaline membranes is primarily responsible for the gas exchange disorders in Covid-19 comprising the hallmark of acute respiratory distress syndrome [36], in autopsy samples of lung, heart, kidney and brain tissues from patients died of Covid-19, systemic endothelial dysfunction was the main finding responsible of regional microthrombosis of the lung vessels, deep vein thrombosis and pulmonary embolism, as well as for the multi-organ dysfunction and failure (kidney failure and cardiac insufficiency), important predictor of mortality [37].

Regarding inflammations molecules, increased expression of vascular and inflammatory factors [such as vascular cell adhesion molecule (VCAM)-1, interleukin (IL)-6, IL-8, monocyte-chemoattractant protein (MCP)-1, E-selectin, TNF-α] were observed in Covid-19 lung tissue, as well as upregulation of ICAM-1, vWF and VEGFR2, and related increased extravasation of inflammatory cells [38]. Interestingly, several studies have confirmed only weak associations with systemic inflammatory parameters such as C-reactive protein, whereas D-dimer levels are strongly correlate with soluble vWF, and other prothrombotic parameters as increased partial thromboplastin time, ICAM-1, E-selectin [39]. In retrospective studies on Covid-19 patients admitted in ICU, management of anticoagulation therapy based on D-dimer was associated with a significant reduction in mortality and renal failure events [40, 41].

In this study, we have found that the Improve risk score and D-Dimer values are related to the development of thromboembolic complications even at a distance after recovery from Covid-19.

Chioh [42] found higher levels of circulating endothelial cells (CECs), dysfunctional endothelial cells arising from damaged vessels, hence representing a surrogate marker of vascular injury and endothelial activation, in patients with previous COVID-19 infection compared to healthy controls; CECs behaviors were related to common cardiovascular risk factors such as hypertension, diabetes and hyperlipidemia. Moreover, CECs showed higher expression of several molecules as ICAM1, SELP, and CX3CL1, expressions of proinflammatory and procoagulant state of the endothelial cells, confirm the vicious cycle caused by vascular damage, activation of coagulation pathway, and inflammation, remained heightened post-Covid-19 infection [43].

The present study is certainly limited by the small number of patients enrolled in a single center and the lack of data regarding the prevalence of PE in overall patients discharged for Covid-19 infection. Therefore, our study may represent a further stimulus to carry out a perspective study to evaluate prevalence of PE on patients discharged from hospital and possible benefits by administration of anticoagulant therapy.

In conclusion, acute Covid-19 infection induces a complex condition affecting not just the lung parenchyma but also other organs; endothelial damage is a crucial characteristic present in all pathophysiological alterations; thromboembolic complications can be detected both acutely and after discharge from hospital. All these complications and pathophysiological modifications can be enhanced in the presence of metabolic and cardiovascular diseases as diabetes.

Different studies show that in a well-defined high-risk group of patients who have been infected with Covid-19, a persistent inflammatory and prothrombotic state remains after recovery; a vascular marker such as sST2 may be useful in determining this inflammatory-prothrombotic state, as well as PCR and D-Dimer. Therefore, the need to evaluate the possible utility of anticoagulant therapy, even in the short term, is of great importance, especially for those who have needed hospitalization or have important concomitant pathologies.
